# Supplementary material for: Stereotactic Body Radiotherapy (SBRT) for the Treatment of Primary Localized Renal Cell Carcinoma: A Systematic Review and Meta-Analysis
Source: Cancers (Basel). 2024 Sep 26;16(19):3276. doi: 10.3390/cancers16193276 (PMC11475739; doi:10.3390/cancers16193276)
Supplement: Supplementary file 1 [file cancers-16-03276-s001.zip › Supplementary File S2 - Research question.pdf]

Supplementary File S2: Population, Intervention, Control, Outcome, Study Design (PICOS) framework used to develop the research question.

Population	Patients treated with primary definitive stereotactic body radiotherapy (SBRT) for localized renal cell carcinoma (RCC).
Intervention	SBRT to the primary tumor (fraction dose ≥5 Gy).
Control	Not applicable (single arm trials) or any standard-of-care treatment
Outcome	Primary: local control, preservation of renal function<br><br>Secondary: progression-free survival; overall survival; rates of grade ≥3 AEs
Study design	Any original prospective studies describing clinical outcomes of patients treated with SBRT for primary RCC
