# Supplementary material for: Stereotactic Body Radiotherapy (SBRT) for the Treatment of Primary Localized Renal Cell Carcinoma: A Systematic Review and Meta-Analysis
Source: Cancers (Basel). 2024 Sep 26;16(19):3276. doi: 10.3390/cancers16193276 (PMC11475739; doi:10.3390/cancers16193276)
Supplement: Supplementary file 1 [file cancers-16-03276-s001.zip › Supplementary File S3 - Search strategy.pdf]

Supplementary File S - Search strategy:

Database(s): MEDLINE, PubMed Central, and Bookshelf (via PubMed platform)

Executed on 10/08/2023

Filters: date of publication 01/01/2003 to 21/07/2023; no language restrictions. The number of results reported in the table includes the use of filters.

#	Pubmed	# hits
#1	(kidney neoplasms[MeSH Terms]) OR (“renal cancer”[TIAB:~2]) OR (“renal carcinoma”[TIAB:~2]) OR (“kidney cancer”[TIAB:~2]) OR (“kidney carcinoma”[TIAB:~2) NOT (wilms[TIAB])	63671
#2	(radiotherapy[MeSH Terms]) OR (proton therapy[MeSH]) OR (radiotherap*[TIAB]) OR (radiation[TIAB]) OR (stereotactic[TIAB]) OR (radiosurg*[TIAB]) OR (cyberknife[TIAB]) OR (volumetric modulated arc therapy[TIAB]) OR (tomotherapy[TIAB]) OR (proton*[TIAB]) OR (carbon-ion[TIAB]) OR (particle therapy [TIAB]) OR (SABR[TIAB]) OR (SBRT[TIAB]) OR (SRS[TIAB])	555263
#3	(primary[TIAB]) OR (definitive[TIAB]) OR (local*[TIAB]) OR (inoperable[TIAB])	2479081
#4	(metasta*[TIAB]) OR (oligometasta*[TIAB])	479463
#5	(non-metasta*[TIAB])	7603
#6	(#1 AND #2 AND #3 NOT #4) OR (#1 AND #2 AND #5)	384

The search strategy was updated on 09/04/2024.

Filters: date of publication 22/07/2023 to 08/04/2024; no language restrictions. The number of results reported in the table includes the use of filters.

#	Pubmed	# hits
#1	(kidney neoplasms[MeSH Terms]) OR (“renal cancer”[TIAB:~2]) OR (“renal carcinoma”[TIAB:~2]) OR (“kidney cancer”[TIAB:~2]) OR (“kidney carcinoma”[TIAB:~2) NOT (wilms[TIAB])	3590
#2	(radiotherapy[MeSH Terms]) OR (proton therapy[MeSH]) OR (radiotherap*[TIAB]) OR (radiation[TIAB]) OR (stereotactic[TIAB]) OR (radiosurg*[TIAB]) OR (cyberknife[TIAB]) OR (volumetric modulated arc therapy[TIAB]) OR (tomotherapy[TIAB]) OR (proton*[TIAB]) OR (carbon-ion[TIAB]) OR (particle therapy [TIAB]) OR (SABR[TIAB]) OR (SBRT[TIAB]) OR (SRS[TIAB])	30197
#3	(primary[TIAB]) OR (definitive[TIAB]) OR (local*[TIAB]) OR (inoperable[TIAB])	160807

#4	(metasta*[TIAB]) OR (oligometasta*[TIAB])	29401
#5	(non-metasta*[TIAB])	521
#6	(#1 AND #2 AND #3 NOT #4) OR (#1 AND #2 AND #5)	30

Database(s): Embase (via Embase platform)

Executed on 10/08/2023

Filters: added to Embase 01/01/2003 to 21/07/2023, records limited to Embase, no language restrictions. The number of results reported in the table includes the use of filters.

#	Embase	# hits
	AND [embase]/lim AND [01-01-2003]/sd NOT [21-07-2023]/sd	n/a
#1	'kidney neoplasms'/exp OR (renal NEAR/2 cancer*):ti,ab OR (renal NEAR/2 carcinoma*):ti,ab OR (kidney NEAR/2 cancer*):ti,ab OR (kidney NEAR/2 carcinoma*):ti,ab	131833
#2	'radiotherapy'/exp OR 'proton therapy'/exp OR radiotherap*:ti,ab OR radiation:ti,ab OR stereotactic:ti,ab OR radiosurg*:ti,ab OR cyberknife:ti,ab OR ‘volumetric modulated arc therapy’:ti,ab OR tomotherapy:ti,ab OR proton*:ti,ab OR 'carbon ion':ti,ab OR ‘particle therapy’:ti,ab OR sabr:ti,ab OR sbrt:ti,ab OR srs:ti,ab	811829
#3	primary:ti,ab OR definitive:ti,ab OR local*:ti,ab OR inoperable:ti,ab	2989731
#4	metasta*:ti,ab OR oligometasta*:ti,ab	679304
#5	non-metastatic:ti,ab	13391
#6	('kidney neoplasms'/exp OR ((renal NEAR/2 cancer*):ti,ab) OR ((renal NEAR/2 carcinoma*):ti,ab) OR ((kidney NEAR/2 cancer*):ti,ab) OR ((kidney NEAR/2 carcinoma*):ti,ab)) AND ('radiotherapy'/exp OR 'proton therapy'/exp OR radiotherap*:ti,ab OR radiation:ti,ab OR stereotactic:ti,ab OR radiosurg*:ti,ab OR cyberknife:ti,ab OR 'volumetric modulated arc therapy':ti,ab OR tomotherapy:ti,ab OR proton*:ti,ab OR 'carbon ion':ti,ab OR 'particle therapy':ti,ab OR sabr:ti,ab OR sbrt:ti,ab OR srs:ti,ab) AND (primary:ti,ab OR definitive:ti,ab OR local*:ti,ab OR inoperable:ti,ab) NOT (metasta*:ti,ab OR oligometasta*:ti,ab) OR (('kidney neoplasms'/exp OR ((renal NEAR/2 cancer*):ti,ab) OR ((renal NEAR/2 carcinoma*):ti,ab) OR ((kidney NEAR/2 cancer*):ti,ab) OR ((kidney NEAR/2 carcinoma*):ti,ab)) AND ('radiotherapy'/exp OR 'proton therapy'/exp OR radiotherap*:ti,ab OR radiation:ti,ab OR stereotactic:ti,ab OR radiosurg*:ti,ab OR cyberknife:ti,ab OR 'volumetric modulated arc therapy':ti,ab OR tomotherapy:ti,ab OR proton*:ti,ab OR 'carbon ion':ti,ab OR 'particle therapy':ti,ab OR sabr:ti,ab OR sbrt:ti,ab OR srs:ti,ab) AND 'non metastatic':ti,ab)	1626

The search strategy was updated on 09/04/2024.

Filters: added to Embase 22/07/2023 to 08/04/2024, records limited to Embase, no language restrictions. The number of results reported in the table includes the use of filters.

#	Embase	# hits
	AND [embase]/lim AND [22-07-2023]/sd NOT [08-04-2024]/sd	n/a
#1	'kidney neoplasms'/exp OR (renal NEAR/2 cancer*):ti,ab OR (renal NEAR/2 carcinoma*):ti,ab OR (kidney NEAR/2 cancer*):ti,ab OR (kidney NEAR/2 carcinoma*):ti,ab	7962
#2	'radiotherapy'/exp OR 'proton therapy'/exp OR radiotherap*:ti,ab OR radiation:ti,ab OR stereotactic:ti,ab OR radiosurg*:ti,ab OR cyberknife:ti,ab OR 'volumetric modulated arc therapy':ti,ab OR tomotherapy:ti,ab OR proton*:ti,ab OR 'carbon ion':ti,ab OR 'particle therapy':ti,ab OR sabr:ti,ab OR sbrt:ti,ab OR srs:ti,ab	51055
#3	primary:ti,ab OR definitive:ti,ab OR local*:ti,ab OR inoperable:ti,ab	200001
#4	metasta*:ti,ab OR oligometasta*:ti,ab	44977
#5	non-metastatic:ti,ab	963
#6	('kidney neoplasms'/exp OR ((renal NEAR/2 cancer*):ti,ab) OR ((renal NEAR/2 carcinoma*):ti,ab) OR ((kidney NEAR/2 cancer*):ti,ab) OR ((kidney NEAR/2 carcinoma*):ti,ab)) AND ('radiotherapy'/exp OR 'proton therapy'/exp OR radiotherap*:ti,ab OR radiation:ti,ab OR stereotactic:ti,ab OR radiosurg*:ti,ab OR cyberknife:ti,ab OR 'volumetric modulated arc therapy':ti,ab OR tomotherapy:ti,ab OR proton*:ti,ab OR 'carbon ion':ti,ab OR 'particle therapy':ti,ab OR sabr:ti,ab OR sbrt:ti,ab OR srs:ti,ab) AND (primary:ti,ab OR definitive:ti,ab OR local*:ti,ab OR inoperable:ti,ab) NOT (metasta*:ti,ab OR oligometasta*:ti,ab) OR (('kidney neoplasms'/exp OR ((renal NEAR/2 cancer*):ti,ab) OR ((renal NEAR/2 carcinoma*):ti,ab) OR ((kidney NEAR/2 cancer*):ti,ab) OR ((kidney NEAR/2 carcinoma*):ti,ab)) AND ('radiotherapy'/exp OR 'proton therapy'/exp OR radiotherap*:ti,ab OR radiation:ti,ab OR stereotactic:ti,ab OR radiosurg*:ti,ab OR cyberknife:ti,ab OR 'volumetric modulated arc therapy':ti,ab OR tomotherapy:ti,ab OR proton*:ti,ab OR 'carbon ion':ti,ab OR 'particle therapy':ti,ab OR sabr:ti,ab OR sbrt:ti,ab OR srs:ti,ab) AND 'non metastatic':ti,ab)	119

Database(s): Scopus (via Scopus platform)

Executed on 10/08/2023

Filters: date of publication 01/01/2003 to 21/07/2023; no language restrictions. The number of results reported in the table includes the use of filters.

#	Scopus	# hits
#1	TITLE-ABS-KEY(renal W/2 (cancer OR carcinoma) OR kidney W/2 (cancer OR carcinoma) AND NOT (wilms))	63323

#2	TITLE-ABS-KEY(radiotherapy OR "proton therapy" OR radiotherap* OR radiation OR stereotactic OR radiosurg* OR cyberknife OR "volumetric modulated arc therapy" OR tomotherapy OR proton* OR "carbon-ion" OR "particle therapy" OR SABR OR SBRT OR SRS)	1935751
#3	TITLE-ABS-KEY(primary OR definitive OR local* OR inoperable)	5811879
#4	TITLE-ABS-KEY(metasta* OR oligometasta*)	713843
#5	TITLE-ABS-KEY(non-metasta*)	8304
#6	(#1 AND #2 AND #3 AND NOT #4) OR (#1 AND #2 AND #5)	590

Database(s): Scopus (via Scopus platform)

The search strategy was updated on 08/04/2024

Filters: date of publication 22/07/2023 to 08/04/2024; no language restrictions. The number of results reported in the table includes the use of filters.

#	Scopus	# hits
#1	TITLE-ABS-KEY(renal W/2 (cancer OR carcinoma) OR kidney W/2 (cancer OR carcinoma) AND NOT (wilms))	N/A
#2	TITLE-ABS-KEY(radiotherapy OR "proton therapy" OR radiotherap* OR radiation OR stereotactic OR radiosurg* OR cyberknife OR "volumetric modulated arc therapy" OR tomotherapy OR proton* OR "carbon-ion" OR "particle therapy" OR SABR OR SBRT OR SRS)	N/A
#3	TITLE-ABS-KEY(primary OR definitive OR local* OR inoperable)	N/A
#4	TITLE-ABS-KEY(metasta* OR oligometasta*)	N/A
#5	TITLE-ABS-KEY(non-metasta*)	N/A
#6	(#1 AND #2 AND #3 AND NOT #4) OR (#1 AND #2 AND #5)	34

Google Scholar search strategy (top 200 hits retrieved)

Filters: date of publication 2003 to 2023; no language restrictions. The number of results reported in the table includes the use of filters.
Executed on 07/08/2023

#	Google Scholar	# hits
	“renal cancer” | “renal carcinoma” | “kidney cancer” | “kidney carcinoma” “radiotherap*” | “radiation” | “stereotactic” | “radiosurg*” | “SABR” | “SBRT” | “cyberknife” | “proton” | “SRS” “primary” | “definitive” | “local” | “inoperable” | “non-metastatic”	17000
