# Supplementary material for: Stereotactic Body Radiotherapy (SBRT) for the Treatment of Primary Localized Renal Cell Carcinoma: A Systematic Review and Meta-Analysis
Source: Cancers (Basel). 2024 Sep 26;16(19):3276. doi: 10.3390/cancers16193276 (PMC11475739; doi:10.3390/cancers16193276)

Study	Surviving	Total	Proportion	95% C.I.
Ponsky et al. (2015)	15	19	0.79	[0.54; 0.94]
Staehler et al. (2015)	36	40	0.90	[0.76; 0.97]
Siva et al. (2017)	33	33	1.00	[0.89; 1.00]
Funayama et al. (2019)	13	13	1.00	[0.75; 1.00]
Kasua et al. (2019)	7	8	0.88	[0.47; 1.00]
Tetar et al. (2020)	33	36	0.92	[0.78; 0.98]
Grubb et al. (2021)	10	11	0.91	[0.59; 1.00]
Kirste et al. (2022)	7	7	1.00	[0.59; 1.00]
Hannan et al. (2023)	16	16	1.00	[0.79; 1.00]
Lapierre et al. (2023)	11	12	0.92	[0.62; 1.00]
Zarkar et al. (2023)	15	19	0.79	[0.54; 0.94]
Yim et al. (2023)	10	10	1.00	[0.69; 1.00]
Siva et al. (2024)	69	70	0.99	[0.92; 1.00]
**Random effects model**		**294**	**0.95**	**[0.88; 0.98]**

Heterogeneity: $\chi^2_{12} = 8.63$ (p = 0.73)

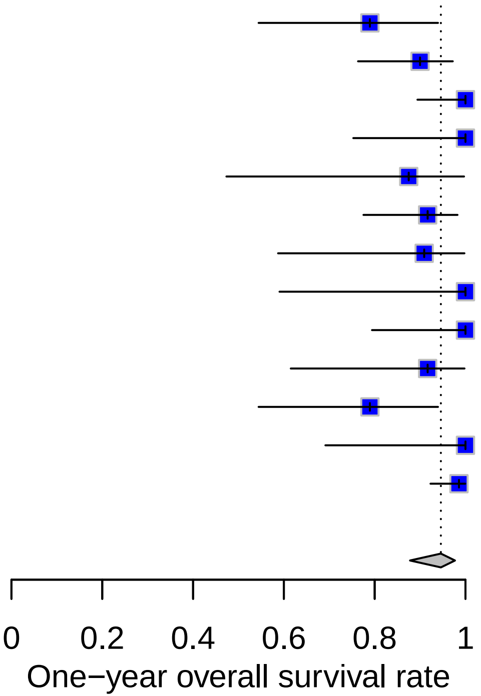

Study	Surviving	Total	Proportion	95% C.I.
Ponsky et al. (2015)	14	19	0.74	[0.49; 0.91]
Staehler et al. (2015)	36	40	0.90	[0.76; 0.97]
Siva et al. (2017)	30	33	0.91	[0.76; 0.98]
Funayama et al. (2019)	12	13	0.92	[0.64; 1.00]
Kasua et al. (2019)	7	8	0.88	[0.47; 1.00]
Tetar et al. (2020)	29	36	0.81	[0.64; 0.92]
Grubb et al. (2021)	9	11	0.82	[0.48; 0.98]
Kirste et al. (2022)	6	7	0.86	[0.42; 1.00]
Hannan et al. (2023)	16	16	1.00	[0.79; 1.00]
Lapierre et al. (2023)	9	12	0.75	[0.43; 0.95]
Zarkar et al. (2023)	12	19	0.63	[0.38; 0.84]
Yim et al. (2023)	10	10	1.00	[0.69; 1.00]
**Random effects model**		**224**	**0.86**	**[0.77; 0.91]**

Heterogeneity: $\chi^2_{11} = 9.90$ (p = 0.54)

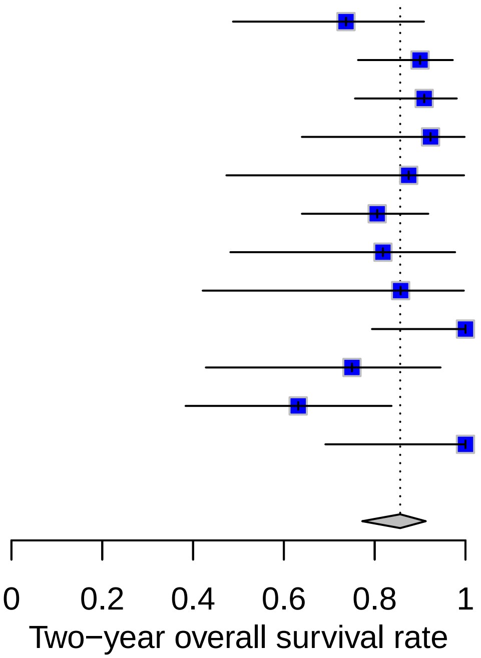

Study	Surviving	Total	Proportion	95% C.I.
Ponsky et al. (2015)	14	19	0.74	[0.49; 0.91]
Staehler et al. (2015)	35	40	0.88	[0.73; 0.96]
Funayama et al. (2019)	9	13	0.69	[0.39; 0.91]
Kasua et al. (2019)	7	8	0.88	[0.47; 1.00]
Tetar et al. (2020)	22	36	0.61	[0.43; 0.77]
Grubb et al. (2021)	7	11	0.64	[0.31; 0.89]
Kirste et al. (2022)	6	7	0.86	[0.42; 1.00]
Hannan et al. (2023)	11	16	0.69	[0.41; 0.89]
Siva et al. (2024)	62	70	0.89	[0.79; 0.95]
**Random effects model**		**220**	**0.78**	**[0.67; 0.86]**

Heterogeneity: $\chi^2_8 = 15.24$ (p = 0.05)

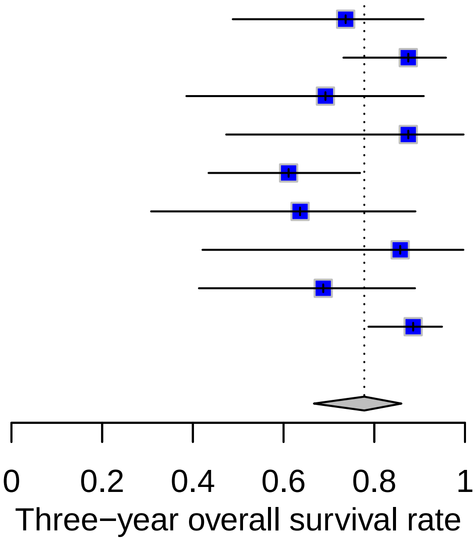

Supplement: Supplementary file 1 [file cancers-16-03276-s001.zip › Supplementary File S5 - Overall survival in the included studies.pdf]
