# Supplementary material for: Stereotactic Body Radiotherapy (SBRT) for the Treatment of Primary Localized Renal Cell Carcinoma: A Systematic Review and Meta-Analysis
Source: Cancers (Basel). 2024 Sep 26;16(19):3276. doi: 10.3390/cancers16193276 (PMC11475739; doi:10.3390/cancers16193276)
Supplement: Supplementary file 1 [file cancers-16-03276-s001.zip › Supplementary File S6 - Funnel plots for G3 AEs, LC, and OS.pdf]

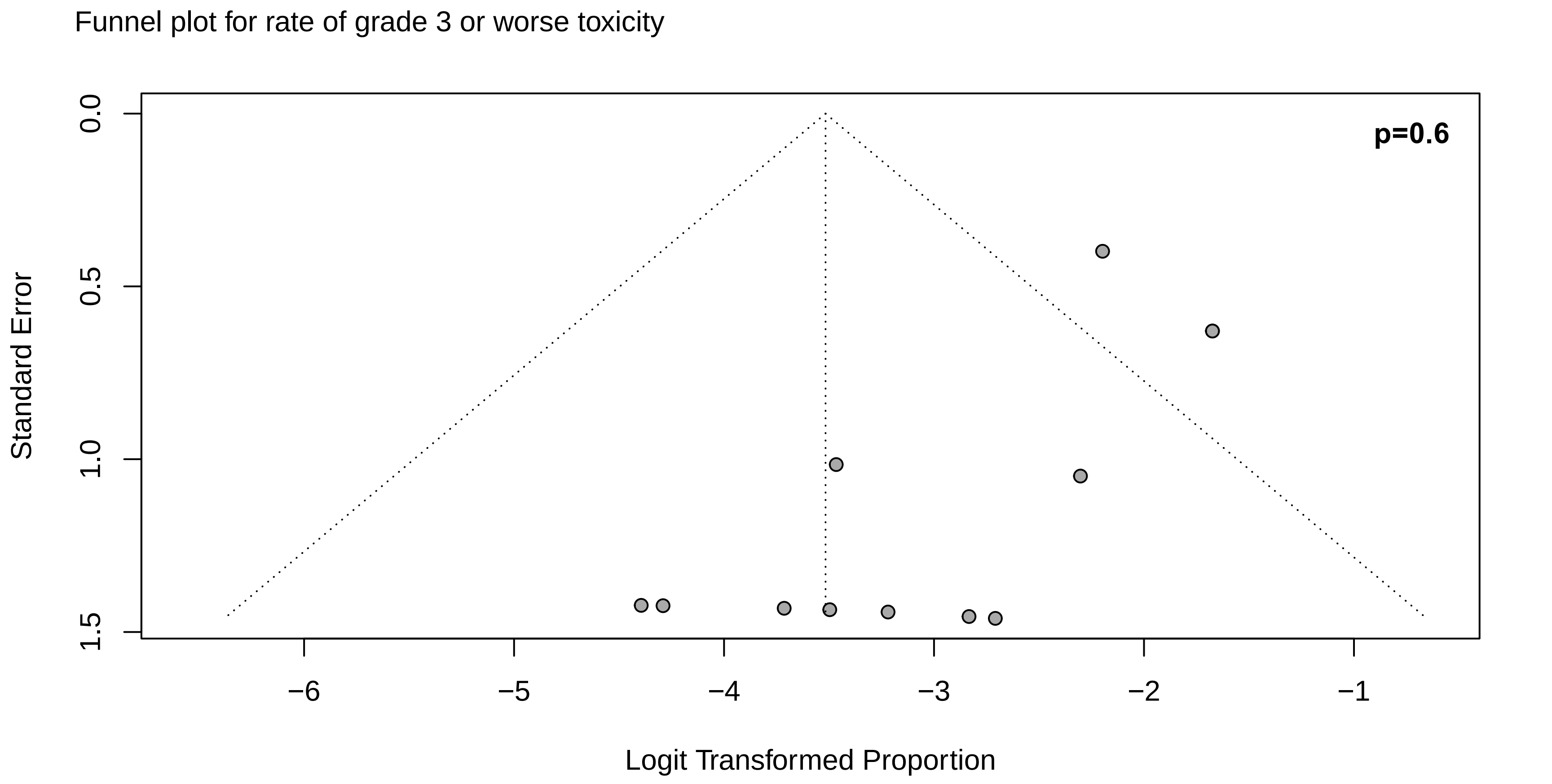

Funnel plot for rate of grade 3 or worse toxicity
p=0.6
Standard Error
0.0
0.5
1.0
1.5
−6
−5
−4
−3
−2
−1
Logit Transformed Proportion

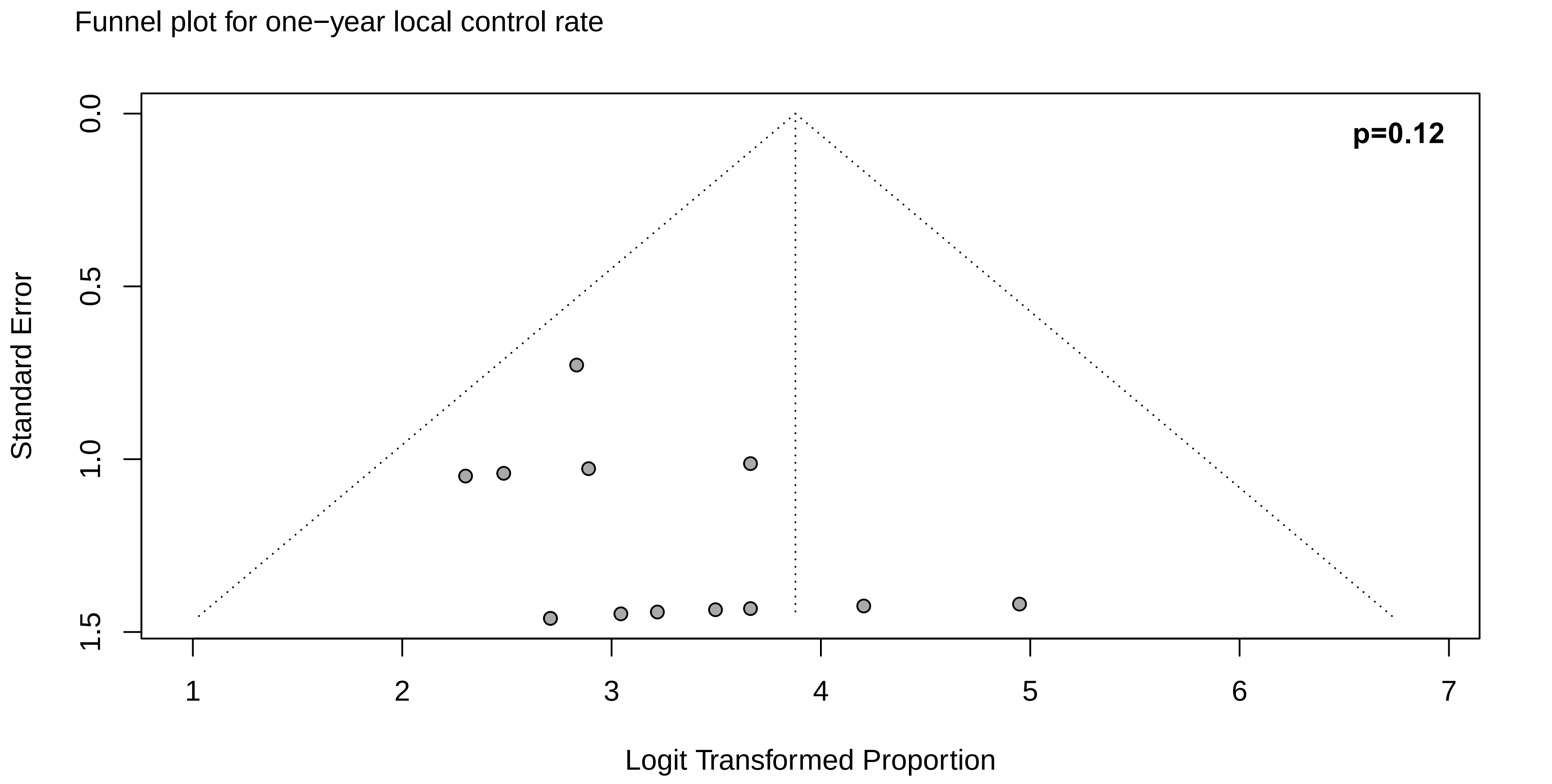
Funnel plot for one−year local control rate
p=0.12
Standard Error
0.0
0.5
1.0
1.5
1
2
3
4
5
6
7
Logit Transformed Proportion

Funnel plot for two−year local control rate

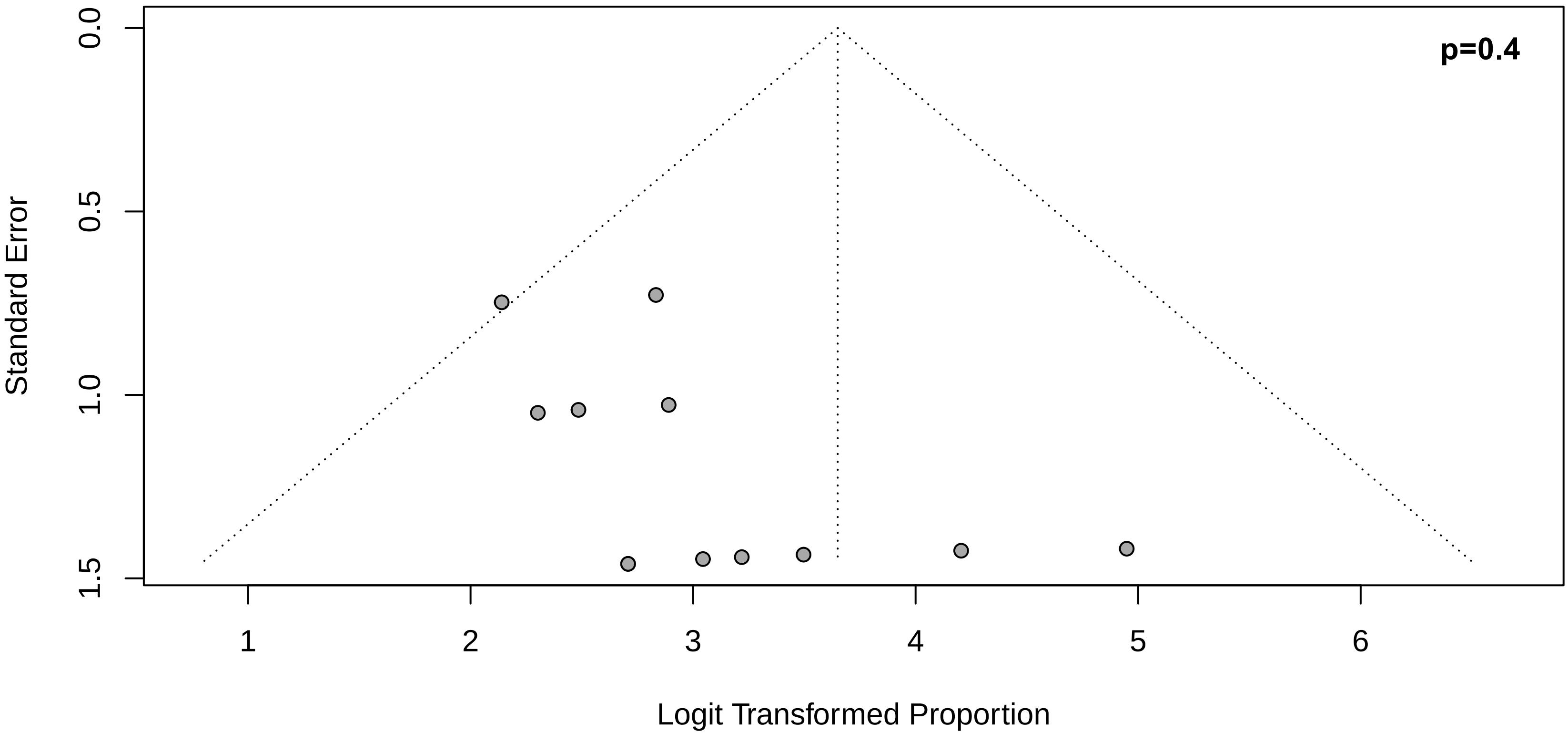

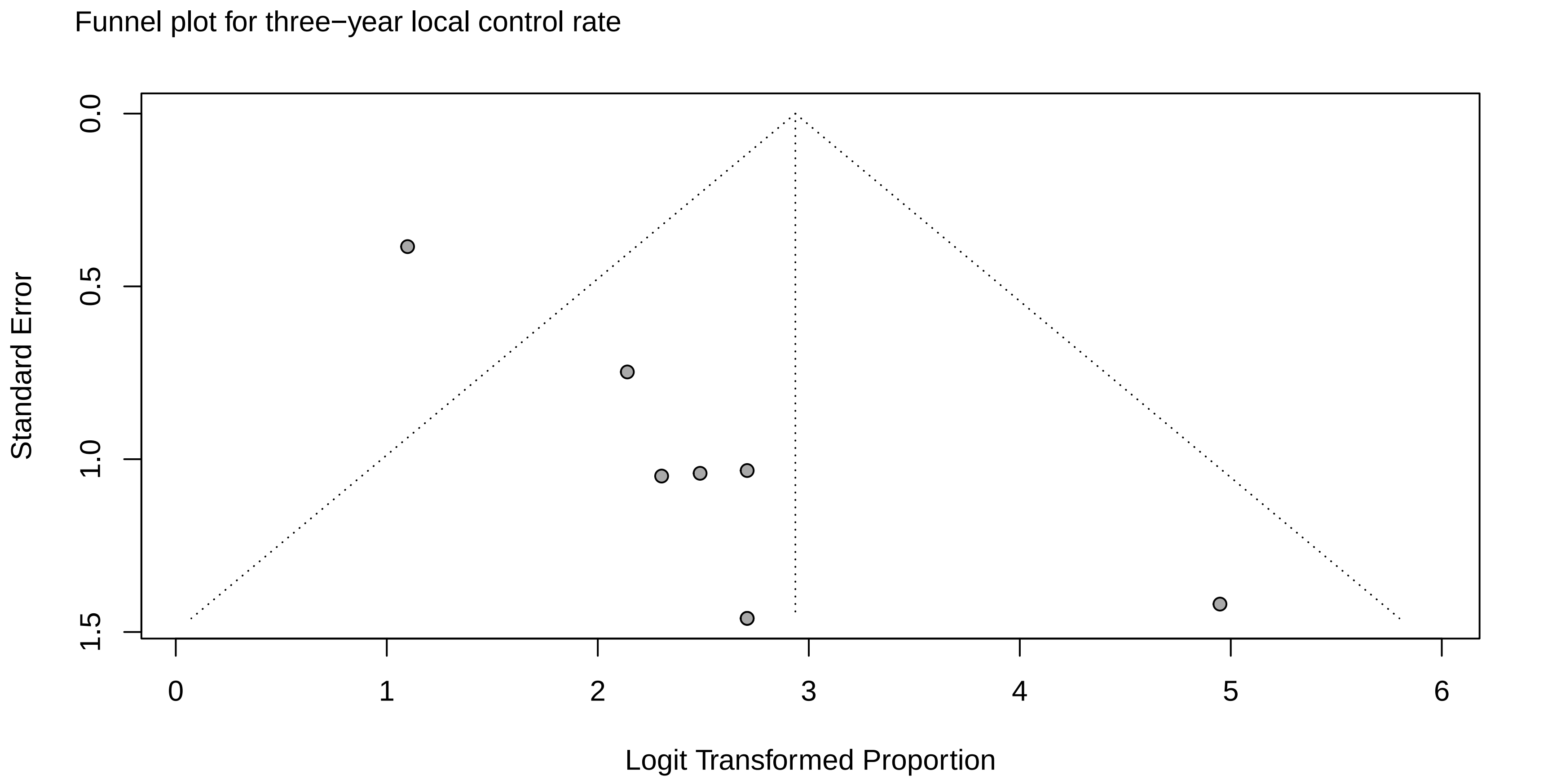

Funnel plot for three−year local control rate
Standard Error
0.0
0.5
1.0
1.5
0
1
2
3
4
5
6
Logit Transformed Proportion

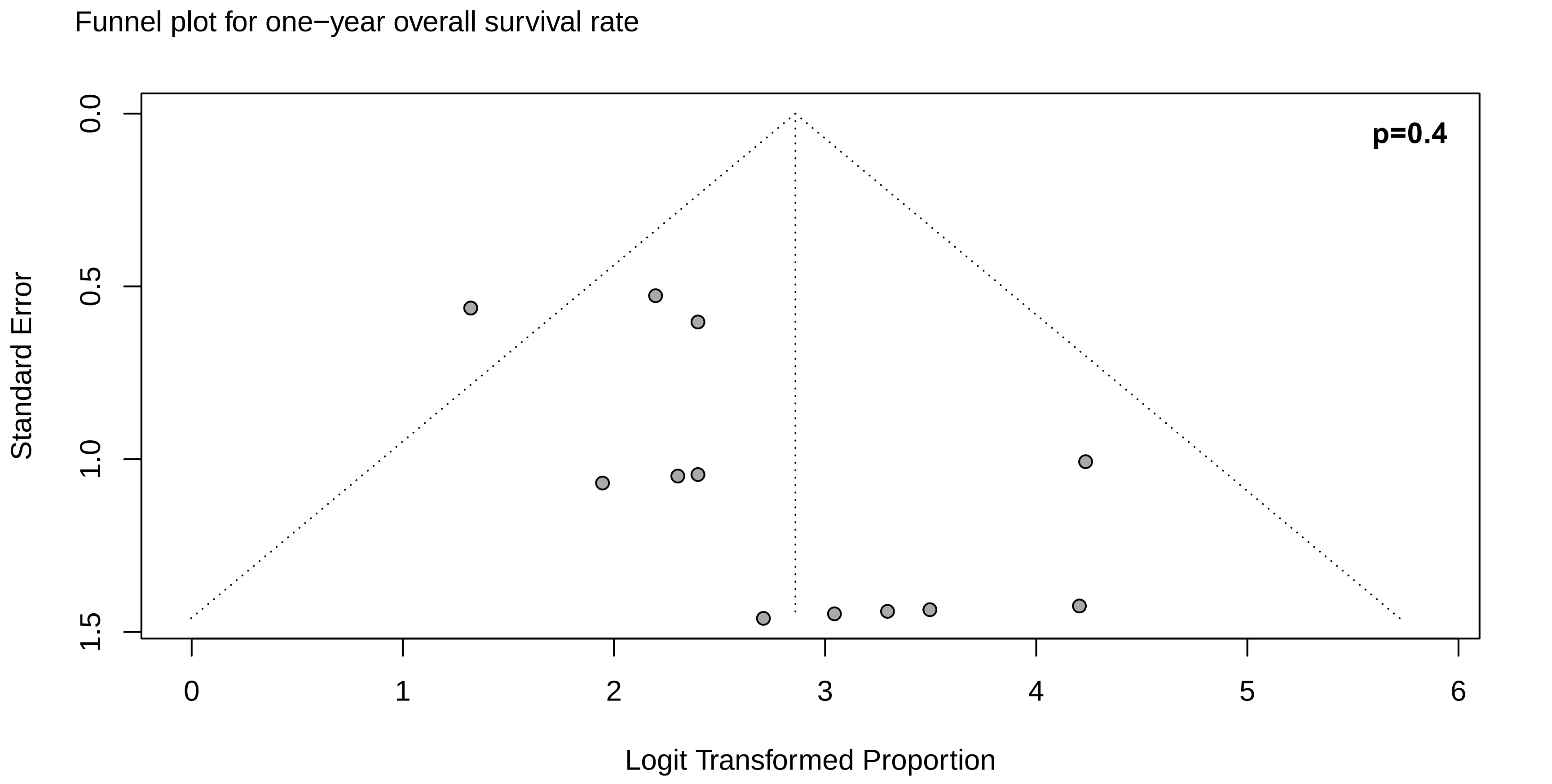

Funnel plot for one−year overall survival rate
p=0.4
Standard Error
0.0
0.5
1.0
1.5
0
1
2
3
4
5
6
Logit Transformed Proportion

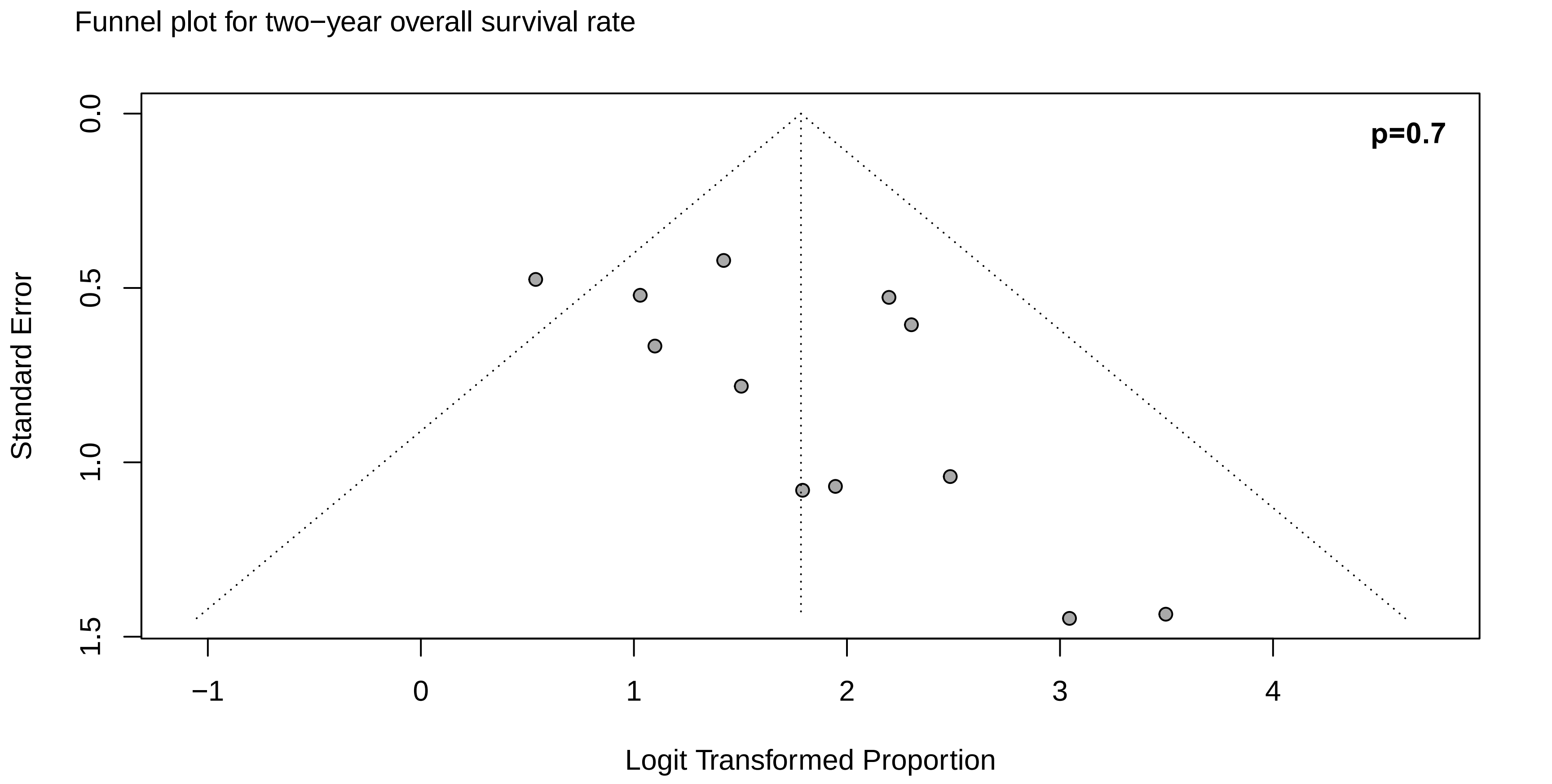

Funnel plot for two−year overall survival rate
p=0.7
Standard Error
0.0
0.5
1.0
1.5
−1
0
1
2
3
4
Logit Transformed Proportion

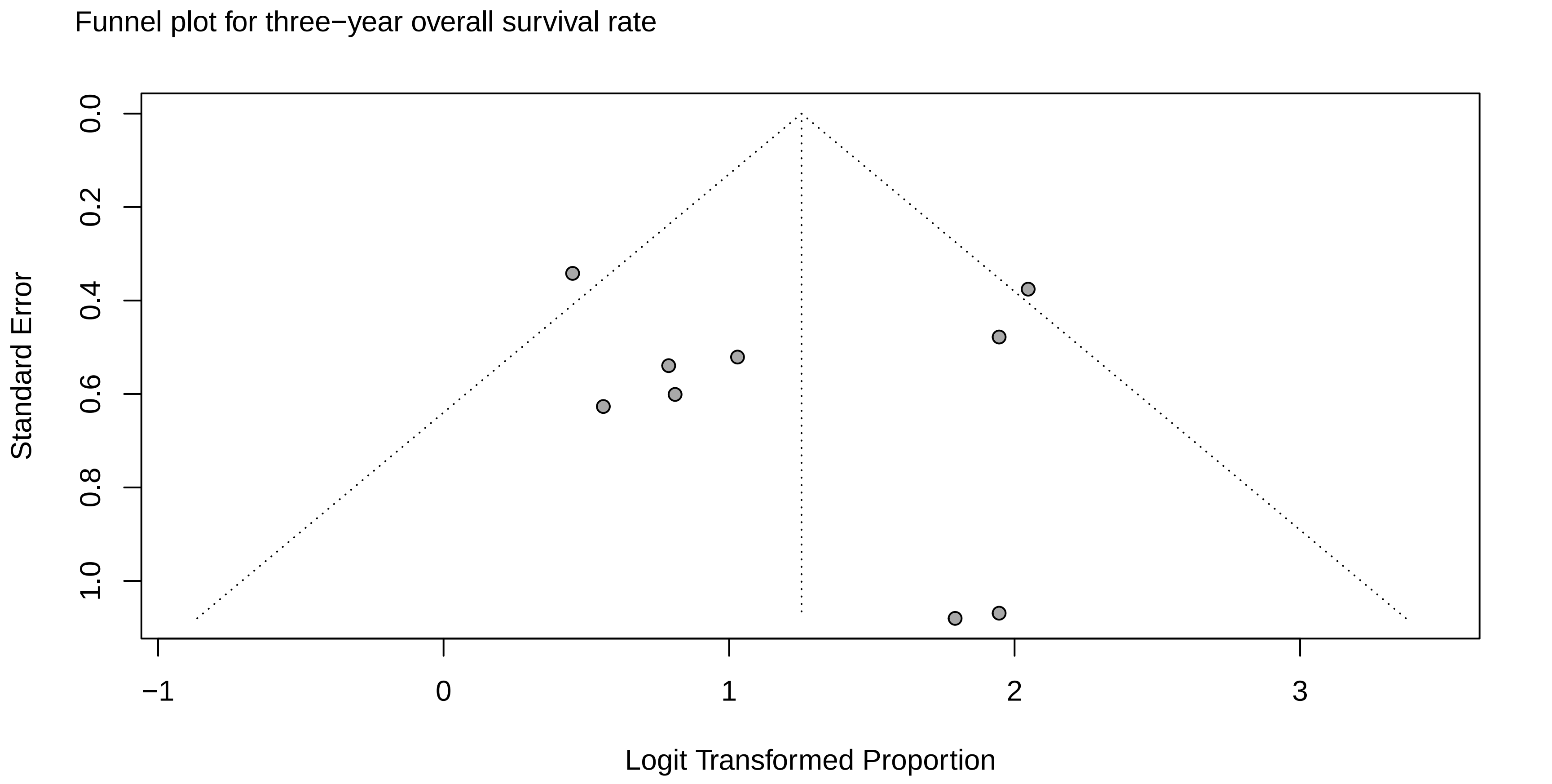
Funnel plot for three−year overall survival rate
Standard Error
0.0
0.2
0.4
0.6
0.8
1.0
−1
0
1
2
3
Logit Transformed Proportion
