# Supplementary material for: Stereotactic Body Radiotherapy (SBRT) for the Treatment of Primary Localized Renal Cell Carcinoma: A Systematic Review and Meta-Analysis
Source: Cancers (Basel). 2024 Sep 26;16(19):3276. doi: 10.3390/cancers16193276 (PMC11475739; doi:10.3390/cancers16193276)

Study	Events	Total	Proportion	95% C.I.
Ponsky et al. (2015)	3	19	0.16	[0.03; 0.40]
Staehler et al. (2015)	0	40	0.00	[0.00; 0.09]
Siva et al. (2017)	1	33	0.03	[0.00; 0.16]
Tetar et al. (2020)	0	36	0.00	[0.00; 0.10]
Grubb et al. (2021)	1	11	0.09	[0.00; 0.41]
Kirste et al. (2022)	0	7	0.00	[0.00; 0.41]
Hannan et al. (2023)	0	16	0.00	[0.00; 0.21]
Lapierre et al. (2023)	0	12	0.00	[0.00; 0.26]
Zarkar et al. (2023)	3	19	0.16	[0.03; 0.40]
Yim et al. (2023)	0	20	0.00	[0.00; 0.17]
Siva et al. (2024)	7	70	0.10	[0.04; 0.20]
**Random effects model**		**283**	**0.03**	**[0.01; 0.12]**

Heterogeneity: $\chi^2_{10} = 2.82$ (p = 0.99)

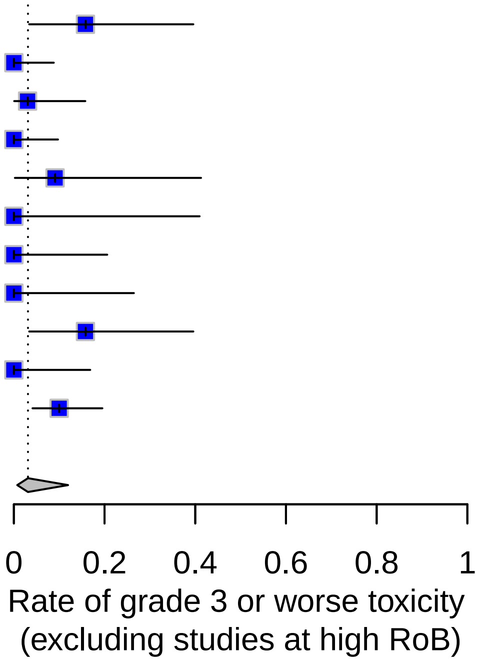

Study	No event	Total	Proportion	95% C.I.
Ponsky et al. (2015)	19	19	1.00	[0.82; 1.00]
Staehler et al. (2015)	39	40	0.97	[0.87; 1.00]
Siva et al. (2017)	33	33	1.00	[0.89; 1.00]
Tetar et al. (2020)	34	36	0.94	[0.81; 0.99]
Grubb et al. (2021)	10	11	0.91	[0.59; 1.00]
Kirste et al. (2022)	7	7	1.00	[0.59; 1.00]
Hannan et al. (2023)	16	16	1.00	[0.79; 1.00]
Lapierre et al. (2023)	12	12	1.00	[0.74; 1.00]
Zarkar et al. (2023)	18	19	0.95	[0.74; 1.00]
Yim et al. (2023)	10	10	1.00	[0.69; 1.00]
Siva et al. (2024)	70	70	1.00	[0.95; 1.00]
**Random effects model**		**273**	**0.98**	**[0.94; 0.99]**

Heterogeneity: $\chi^2_{10} = 0.90$ (p = 1.00)

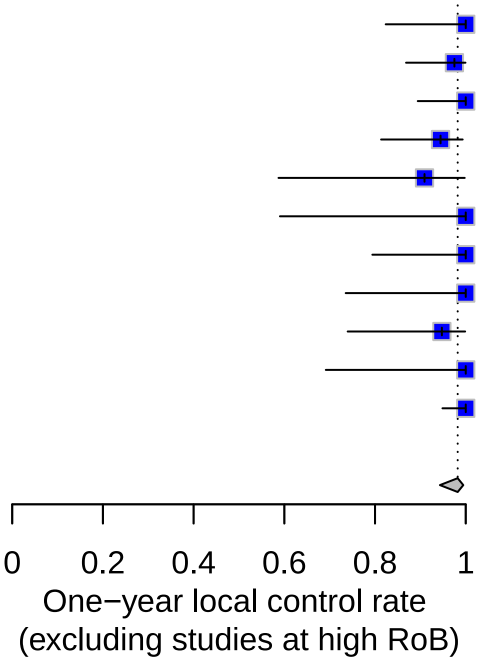

Study	No event	Total	Proportion	95% C.I.
Ponsky et al. (2015)	17	19	0.89	[0.67; 0.99]
Siva et al. (2017)	33	33	1.00	[0.89; 1.00]
Tetar et al. (2020)	34	36	0.94	[0.81; 0.99]
Grubb et al. (2021)	10	11	0.91	[0.59; 1.00]
Kirste et al. (2022)	7	7	1.00	[0.59; 1.00]
Hannan et al. (2023)	16	16	1.00	[0.79; 1.00]
Lapierre et al. (2023)	12	12	1.00	[0.74; 1.00]
Zarkar et al. (2023)	18	19	0.95	[0.74; 1.00]
Yim et al. (2023)	10	10	1.00	[0.69; 1.00]
Siva et al. (2024)	70	70	1.00	[0.95; 1.00]
**Random effects model**		**233**	**0.98**	**[0.92; 0.99]**

Heterogeneity: $\chi^2_9 = 0.62$ (p = 1.00)

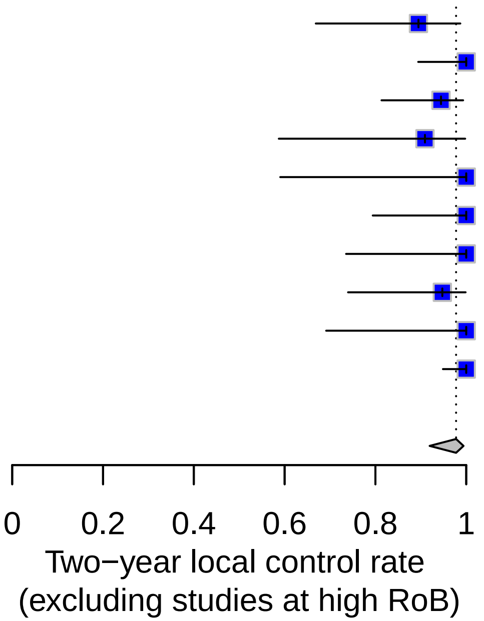

Study	No event	Total	Proportion	95% C.I.
Ponsky et al. (2015)	17	19	0.89	[0.67; 0.99]
Tetar et al. (2020)	27	36	0.75	[0.58; 0.88]
Grubb et al. (2021)	10	11	0.91	[0.59; 1.00]
Kirste et al. (2022)	7	7	1.00	[0.59; 1.00]
Hannan et al. (2023)	15	16	0.94	[0.70; 1.00]
Siva et al. (2024)	70	70	1.00	[0.95; 1.00]
**Random effects model**		**159**	**0.95**	**[0.74; 0.99]**

Heterogeneity: $\chi^2_5 = 3.77$ ($p = 0.58$)

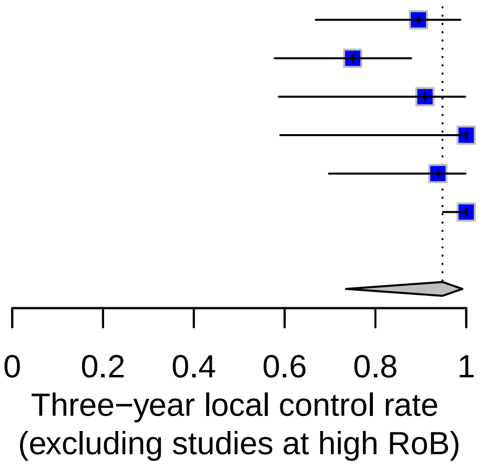

Supplement: Supplementary file 1 [file cancers-16-03276-s001.zip › Supplementary File S7 - Sensitivity analysis (excluding high RoB) for G3 AEs and LC.pdf]
