# Supplementary material for: Stereotactic Body Radiotherapy (SBRT) for the Treatment of Primary Localized Renal Cell Carcinoma: A Systematic Review and Meta-Analysis
Source: Cancers (Basel). 2024 Sep 26;16(19):3276. doi: 10.3390/cancers16193276 (PMC11475739; doi:10.3390/cancers16193276)
Supplement: Supplementary file 1 [file cancers-16-03276-s001.zip › Supplementary Table S1 - Detailed descriptions of treatment-related adverse events.pdf]

Table S1 – Summary of treatment-related grade ≥3 adverse events in prospective studies evaluating the use of stereotactic ablative radiosurgery (SBRT) in renal cell carcinoma patients.

Publication year, 1st author	N	N patients	CTCAE version	Event description: grade (CTCAE); attribution; CTCAE term and timing (days after SBRT); additional explanation (if applicable)
Ponsky et al. [2015][29]	19	3	4.0	1) G4; possibly related; duodenal ulcer at 44 days and 482 days with maximal dose to the bowel of 54 Gy in 4 fractions<br>2) G3; possibly related; mean eGFR 15 mL/min/1.73 m2<br>3) G3; possibly related; mean eGFR 16 mL/min/1.73 m2
Staehler et al. [2015][25]	40	0	3.0	
Siva et al. [2017][35]	33	1	4.0	1) G3; definitely related; fatigue at >90 days
Funayama et al. [2019][33]	N/A	N/A	4.0	
Kasuya et al. [2019][34]	8	0	4.0	
Tetar et al. [2020][26]	36	0	4.0	
Grubb et al. [2021][30]	11	1	4.0	1) G3; possibly related; acute pyelonephritis at 2 years
Kirste et al. [2022][27]	7	0	4.0	
Hannan et al. [2023][31]	16	0	4.0	
Lapierre et al. [2023][21]	12	0	4.0	
Zarkar et al. [2023][28]	19	3	4.0	1) G3; NA; colitis at 6 weeks<br>2) G3; NA; haematuria at 6 weeks<br>3) G3; NA; anaemia at 9 months
Yim et al. [2023][32]	20	0	5.0	

Siva et al. [2024]^[36]	70	7	4.0	1) G3; NS*; vomiting<br>2) G3; NS*; vomiting<br>3) G3; NS*; abdominal pain<br>4) G3; NS*; abdominal pain<br>5) G3; NS*; colonic obstruction<br>6) G3; NS*; colonic obstruction<br>7) G3; NS*; flank pain<br>8) G3; NS*; nausea<br>9) G3; NS*; diarrhoea<br>10) G3; NS*; tumour pain

Abbreviations: SBRT - stereotactic body radiotherapy; N – number of patients; CTCAE – Common Toxicity Criteria for Adverse Events; N/A – not available (assumed as treatment related); eGFR - estimated glomerular filtration rate; ^ - 7 patients, 10 events; NS – not specified if possibly/probably/ definitely treatment related
