# Supplementary material for: Stereotactic Body Radiotherapy (SBRT) for the Treatment of Primary Localized Renal Cell Carcinoma: A Systematic Review and Meta-Analysis
Source: Cancers (Basel). 2024 Sep 26;16(19):3276. doi: 10.3390/cancers16193276 (PMC11475739; doi:10.3390/cancers16193276)
Supplement: Supplementary file 1 [file cancers-16-03276-s001.zip › Supplementary Table S2 - Preservation of kidney function.pdf]

Table S2 – Assessment of renal function following SBRT for the treatment of primary localised renal cell carcinoma

Publication year, 1st author	Baseline median eGFR	When the decline in eGFR was assessed?	N of evaluated patients	eGRF decline
Ponsky et al. [2015][29]	N/A			2 patients with G3 renal toxicity
Staehler et al. [2015][25]	76.8 ml/min	follow-up	N/A	-6.5 ml/min
Siva et al. [2017][35]	55 ml/min (mean)	1 year	33	-11 ml/min (95% CI -17; -6)
		2 years	9	-11 ml/min (95% CI -19; -3)
Funayama et al. [2019][33]	N/A			CKD grade change, increase in CKD in 11 cases, one 4 and one 5
Kasuya et al. [2019][34]	64.1 ml/min	End of follow-up (≥2 years)	6	-10.8 ml/min
Tetar et al. [2020][26]	55.8 ml/min (mean)	End of follow-up (mean 16 months)	36	-6 ml/min
Grubb et al. [2021][30]	82 ml/min	3 years	N/A	-7 ml/min
Kirste et al. [2022][27]	83.7ml/min	6 months	7	-5.3ml/min
		1 year	5	-7.1 ml/min
		2 years	5	-5.2 ml.min
Hannan et al. [2023][31]	61.2 ml/min	1 year	15	-10.2 ml/min (95% CI -16.3; -4.1)
		2 years	10	-4 ml/min (95% CI -8.1; 0.1)
		3 years	8	-12.1 ml/min (95% CI -19.6; -4.6)
		End of follow-up (median 35.4 months)	16	-10.3 ml/min (95% CI -18.3; -2.2)
Lapierre et al. [2023][31]	64 ml/min	End of follow-up	12	-8.7 ml/min (95% CI 2.1-15.3)
Zarkar et al. [2023][21]	62 ml/min	6 months	N/A	-5.4 ml/min (95% CI -10.3; -0.6)
		1 year	N/A	-8.7 ml/min (95% CI -15.3; -2.1)
Yim et al. [2023][28]	44.5 ml/min	End of follow-up	20	-1.8 ml/min (95% CI -6.6; 3.1)
Siva et al. [2024][32]	61.1 ml/min (mean)	1 year	N/A	-10.8 ml/min (-13.1 to -8.5)
		2 years	N/A	-14.6 ml/min (-17.1 to -12.1)

eGFR – estimated glomerular filtration rate; N/A – not avaiable
