# Supplementary figures and images for: Stereotactic Body Radiotherapy (SBRT) for the Treatment of Primary Localized Renal Cell Carcinoma: A Systematic Review and Meta-Analysis
Source: Cancers (Basel). 2024 Sep 26;16(19):3276. doi: 10.3390/cancers16193276 (PMC11475739; doi:10.3390/cancers16193276)

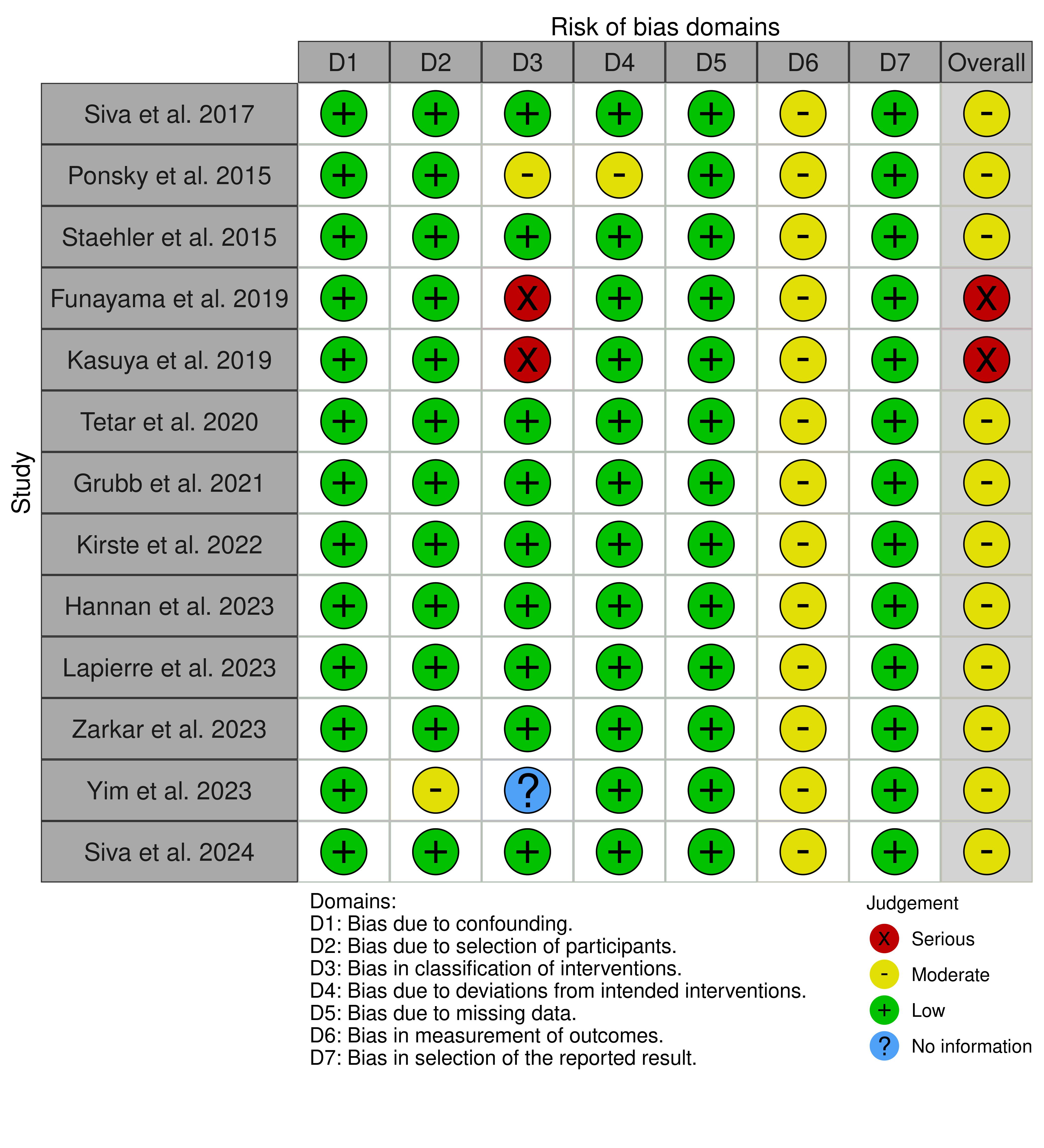

Supplement: Supplementary file 1 [file cancers-16-03276-s001.zip › Supplementary File S4 - Risk of Bias.jpeg]
